# The Complete Chloroplast Genome of *Clematis tangutica (Maxim.) Korsh*. and an Adaptive Evolutionary Analysis of the *ycf2* Gene

**DOI:** 10.3390/genes17080930

**Published:** 2026-08-10

**Authors:** Xuebing Zhu, Xiaozhu Guo, Lihui Wang, Shipeng Yang, Xuemei Sun

**Affiliations:** 1 Academy of Agriculture and Forestry Sciences, Qinghai University, Xining 810016, China; 15297190798@163.com (X.Z.);; 2 Laboratory for Research and Utilization of Germplasm Resources in Qinghai Tibet Plateau, Qinghai University, Xining 810016, China

**Keywords:** adaptive evolution, chloroplast genome, *Clematis tangutica (Maxim.) Korsh*, *ycf2*

## Abstract

Background: *Clematis tangutica (Maxim.) Korsh*. is a Tibetan medicinal plant, but its chloroplast genome and plastid gene evolution remain unexplored. Here, we assembled the complete chloroplast genome of *C. tangutica* and investigated the adaptive evolution of the *ycf2* gene. Methods: Chloroplast DNA was extracted from fresh leaves and sequenced on the Illumina HiSeq PE150 platform. We then performed comprehensive genomic analyses, including genome structure characterization, repeat and SSR identification, comparative genomics, and positive selection analysis of the *ycf2* gene. Results: The genome is 159,584 bp with a typical quadripartite structure, containing 134 genes and 23 SSRs. Comparative analyses revealed that *ycf2* is the most variable gene among the 13 divergent loci identified. Positive selection analysis detected 12 significant sites in *ycf2*, all clustered in the middle region of the protein. Conclusions: This study provides the first complete chloroplast genome resource for *C. tangutica*, offers new insights into the adaptive evolution of *ycf2* in Ranunculaceae, and yields molecular markers applicable to population genetics, phylogenetic studies, and conservation planning for this species of medicinal importance.

## 1. Introduction

The genus Clematis is among the largest in the Ranunculaceae, with approximately 355 species worldwide [1]. China is a major diversity center, harboring 147 species, of which 93 are endemic [2]. Among these, 70 species are used in traditional Chinese medicine, reflecting the genus’s significant medicinal value [3]. Besides their medicinal value, many Clematis species are also cultivated as ornamentals due to their showy flowers and diverse floral morphologies.

As a perennial twining species, *Clematis tangutica (Maxim.) Korsh.* occurs naturally in Tibet, Qinghai, Gansu, and Sichuan provinces [4,5]. In Tibetan medicine, the whole plant, known as “Ye-Mang-Na-Bao,” is used to treat skin ulcers, indigestion, and a range of other ailments including rheumatism, inflammation, and nervous disorders. Pharmacological studies have reported analgesic, anti-inflammatory, antibacterial, and anticancer activities associated with this species [6]. Despite its medicinal and ecological importance, the genetic resources of *C. tangutica* remain largely unexplored.

Understanding the genetic basis of adaptation in this species requires evaluating the evolutionary pressures acting on its chloroplast genes. To infer the mode of selection acting on protein-coding genes, we calculated the ratio of non-synonymous to synonymous substitution rates (ω = dN/dS). Under this framework, ω < 1, ω = 1, and ω > 1 correspond to purifying selection, neutral evolution, and positive selection, respectively [7]. Detecting positive selection in chloroplast genes can thus provide insights into their adaptive potential in response to environmental pressures. Recent studies have increasingly reported positive selection signals in chloroplast genes associated with stress responses and ecological adaptation, suggesting that plastid genomes may be more dynamically involved in plant adaptation than previously thought.

Chloroplasts, the organelles responsible for converting light energy into chemical energy via photosynthesis, play a central role in plant growth and metabolism [8]. They possess their own genome, which has become a powerful tool for evolutionary and phylogenetic studies due to its maternal inheritance, relatively conserved structure, and manageable size [9,10]. Over the past decade, chloroplast genome sequencing has yielded significant insights into plant evolution and adaptation [11]. For instance, comparative plastid genomics has resolved complex phylogenetic relationships in taxonomically challenging groups such as *Passiflora* [12], and has revealed adaptive evolution of genes involved in photosynthetic efficiency in response to environmental stress in *Rutaceae* [13,14]. More importantly, positive selection has been detected in chloroplast genes linked to environmental adaptation [15]. For example, the *ndhF* gene in a widely distributed orchid lineage exhibited an elevated Ka/Ks ratio of 3.65, suggesting adaptive evolution in response to heterogeneous habitats [16]. Despite the medicinal and ecological importance of *C. tangutica*, its genomic data remain unavailable, so we conducted a comprehensive analysis of its chloroplast genome to characterize its genetic architecture and provide a foundation for exploring its medicinal potential.

As the largest open reading frame in the chloroplast genome, *ycf2* encodes a protein of more than 2200 amino acids and has been shown to be essential for cell survival, as knockout mutants cannot not be maintained in a homoplastomic state [17,18]. Recent structural studies have further identified *ycf2* as the core ATPase motor of the chloroplast protein import machinery, highlighting its fundamental role in plastid biogenesis [18,19]. Beyond its essential function, *ycf2* has also attracted considerable attention as a hotspot of adaptive evolution. Positive selection signals have been detected in *ycf2* across multiple angiosperm lineages: 14 positive selection sites were identified in the orchid genus *Holcoglossum* [20], several sites were found in *Asteraceae* [21], and additional sites were reported in *Chrysosplenium* within Saxifragaceae [22]. These findings suggest that *ycf2* may have experienced lineage-specific adaptive evolution in response to diverse environmental pressures. However, no study has yet investigated the evolutionary dynamics of *ycf2* in the Ranunculaceae family, which includes species adapted to a wide range of habitats, from temperate to high-altitude environments. To fill this gap, we employed Illumina sequencing to assemble and annotate the complete chloroplast genome of *C. tangutica*. We then performed comparative genomic analyses and codon-based positive selection tests on the *ycf2* gene. Here, we report the first chloroplast genome resource for *C. tangutica*, offering valuable genetic information for future studies on its medicinal properties, population genetics, and conservation.

## 2. Materials and Methods

### 2.1. Chloroplast DNA Extraction and Sequencing

Leaf samples of *Clematis tangutica (Maxim.) Korsh.* were obtained from the Qinghai University, Xining, China. Chloroplast DNA was extracted following a previously described high-throughput method [23]. An Illumina HiSeq PE150 instrument was used to sequence a paired-end library with an average insert size of 150 bp.

### 2.2. Chloroplast Genome Assembly and Annotation

FastQC v0.11.9 performed quality filtering, and low-quality reads were removed before assembly. Initial contig assembly was carried out using SOAPdenovo v2.04 with default parameters to generate initial contigs [24]. The preliminary contigs were then used as input for final assembly with SPAdes v3.14.1 using default k-mer settings and the --careful option enabled to reduce assembly errors, without applying a coverage cut-off [25].

To identify chloroplast-derived contigs, the assembled scaffolds were subjected to BLASTn(v2.13.0) (e-value ≤ 1 × 10^−10^) and Exonerate (protein similarity ≥ 70%) searches against previously reported chloroplast genome sequences and protein-coding gene datasets of related species. Scaffolds matching chloroplast sequences were selected and ranked by coverage depth; contigs with significantly lower coverage compared to the majority of chloroplast contigs were discarded as potential nuclear or mitochondrial contaminants.

The selected chloroplast scaffolds were merged and further extended using PRICE (v1.0) with a paired-end insert size of 300 bp (estimated total fragment length of 600 bp for 150 bp paired-end reads), minimum read-to-contig similarity of 95%, minimum overlap length of 30 bp, minimum read identity of 90%, and 50 iterations to minimize scaffold number. To orient the assembled contigs and define the IR/SC boundaries, the chloroplast genome of *Clematis aethusifolia* (GenBank accession MK253462) was used as a reference. The extended sequences were then used for iterative reference-based assembly: sequencing reads were mapped to the extended scaffolds using Bowtie2 v2.4.5 with the -N 1 parameter, and the mapped read pairs were extracted and reassembled with SPAdes using optimized k-mer values (93, 95, 97, 103, 105, 107, 115) to improve assembly continuity. The assembly was iterated until the genome reached a circular conformation.

Genome annotation was carried out using PAG [26], with all parameters set to default. Protein-coding gene boundaries were manually verified. tRNAscan-SE v2.0 was employed for tRNA gene prediction [27], and rRNA genes were predicted with RNAmmer v1.2 under default settings. The chloroplast genome was visualized as a circular map using OGDRAW v1.3.1 [28].

### 2.3. Repeats and SSRs Analysis

Dispersed repeats (≥30 bp, ≥90% similarity) were detected using REPuter. Tandem repeats were found with Tandem Repeats Finder v4.09 (match 2, mismatch 7, indel 7; min score 70; max period 1000; max array 5 Mb). SSRs were detected using Krait v1.3.3, with minimum repeat units of 12/7/5/4/4/4 for mono-/di-/tri-/tetra-/penta-/hexa-nucleotides and motif standardization at level 3.

### 2.4. Comparative Chloroplast Genome Analysis

Pairwise genome alignments between *C. tangutica* and eight other species, *C. tangutica* (MK253446.1), *C. uncinata* (NC_039846.1), *C. guniuensis* (NC_050373.1), *Paeonia suffruticosa* (OK662586.1), *P. mlokosewitschii* (NC_071855.1), *Trollius macropetalus* (NC_059918.1), *T. farreri* (NC_050872.1), and *Anemone nemorosa* (NC_066446.1) were generated using the LAGAN model in mVISTA [29], IR boundaries were plotted with IRscope [30], and a Circos map was created with HomBlocks [31] to display gene distribution, with blocks colored by sequence length (red, orange, green, and blue for long, medium, short, and very short fragments). The *ycf2* protein alignment was performed by COBALT ((https://www.ncbi.nlm.nih.gov/tools/cobalt/cobalt.cgi) accessed on 10 August 2022). All tools used their default settings.

### 2.5. Phylogenetic Inference

Phylogenetic reconstruction employed 26 chloroplast genomes, comprising 15 Ranunculaceae species (*Clematis*, *Trollius*, *Anemone*) and 11 outgroup taxa (*Paeoniaceae*, *Brassicaceae*, *Solanaceae*, *Asteraceae*). MAFFT v7.490 [32] was used for sequence alignment, and the optimal substitution model was selected with jModelTest v2.1.7 under BIC. Phylogenetic reconstruction employed IQ-TREE v8.1.24 [33] (1000 bootstrap replicates) for ML analysis and MrBayes v3.2 [34] for Bayesian inference under GTR + I + G model (four chains, 5 × 10^6^ generations, sampled every 500 trees, burn-in = 20%).

### 2.6. Positive Selection Analysis of the ycf2 Gene

Positive selection analysis of the *ycf2* gene was performed using the codon-based models implemented in EasyCodeML [35]. The following model pairs were tested: M1a–M2a, M0–M3, and M7–M8. Likelihood ratio tests (LRTs) were conducted using PAMLx V1.3.1 [36] to evaluate model fit, with significance determined using a χ^2^ distribution. Naïve Empirical Bayes (NEB) and Bayes Empirical Bayes (BEB) were applied to detect positively selected sites, requiring a posterior probability >95%.

## 3. Results

### 3.1. Genome Organization and Gene Features

**Figure 1:**
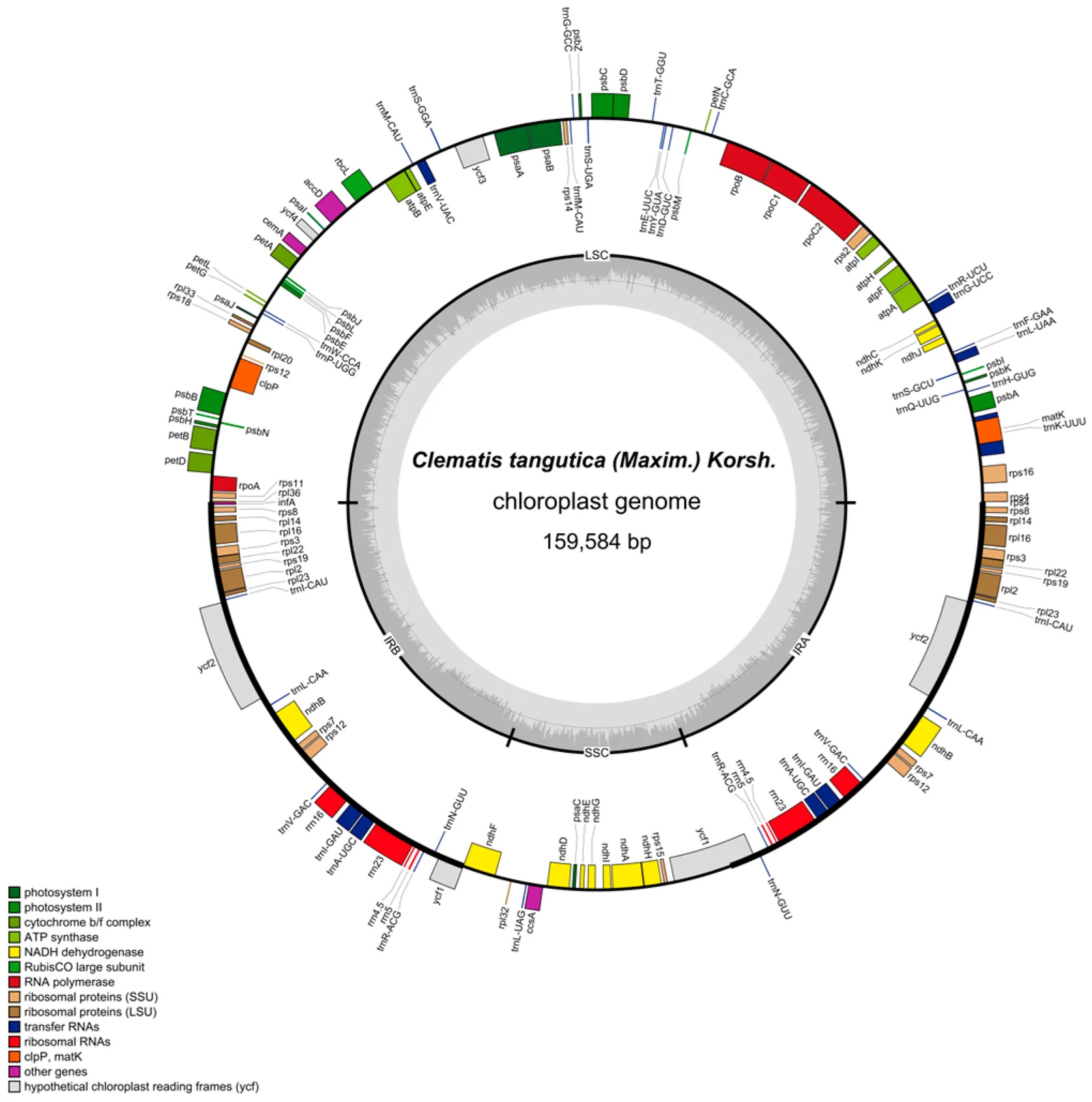
Gene map of the *C. tangutica* chloroplast genome. Outer circle genes are transcribed clockwise; inner circle genes counter-clockwise. Colors denote functional categories (legend, bottom left). The LSC, SSC, and two IR regions are labeled. GC content is shown by the inner gray ring, with the 50% threshold marked by the middle line.

A total of 4.39 Gb of raw reads were generated, providing 972× coverage of the chloroplast genome. The assembled genome of *C. tangutica* is 159,584 bp, with the classic quadripartite organization (LSC, SSC, and two IRs) of 79,515 bp, 17,985 bp, and 31,042 bp, respectively. The overall GC content is 37.9%, with the IR (42.0%) substantially higher than the LSC (36.3%) and SSC (31.4%). Total of 134 genes were annotated, comprising 88 protein-coding, 38 tRNA, and 8 rRNA genes (Table 1; Figure 1; Table S1). Among these, 16 genes (eight protein-coding, six tRNA, and two rRNA) are located in the IRs and transcribed in the reverse direction. The protein-coding genes include *ycf2*, *ndhB*, *rps2*, *rps3*, *rps12*, *rpl2*, *rpl22*, and *rpl23*; the rRNA genes are *rrn16* and *rrn23*. Seventeen intron-containing genes were identified (11 protein-coding and 6 tRNA), with the longest intron in *trnK-UUU* (2545 bp) and the shortest in *petB* (483 bp). The *clpP* and *ycf3* genes contain two introns each, while all others contain a single intron (Table S2). The *rps12* gene is trans-spliced.

### 3.2. Repeat Sequences and SSR Markers

Detection of 26 dispersed repeats (≥30 bp, Hamming distance = 3) were identified, including 14 forward (F), 8 reverse complement (P), 3 reverse (R), and 1 complement (C). Ten tandem repeats (9–24 bp) were also identified, with two in LSC, three in SSC, and five in Irs (three each), each occurring twice.

**Figure 2:**
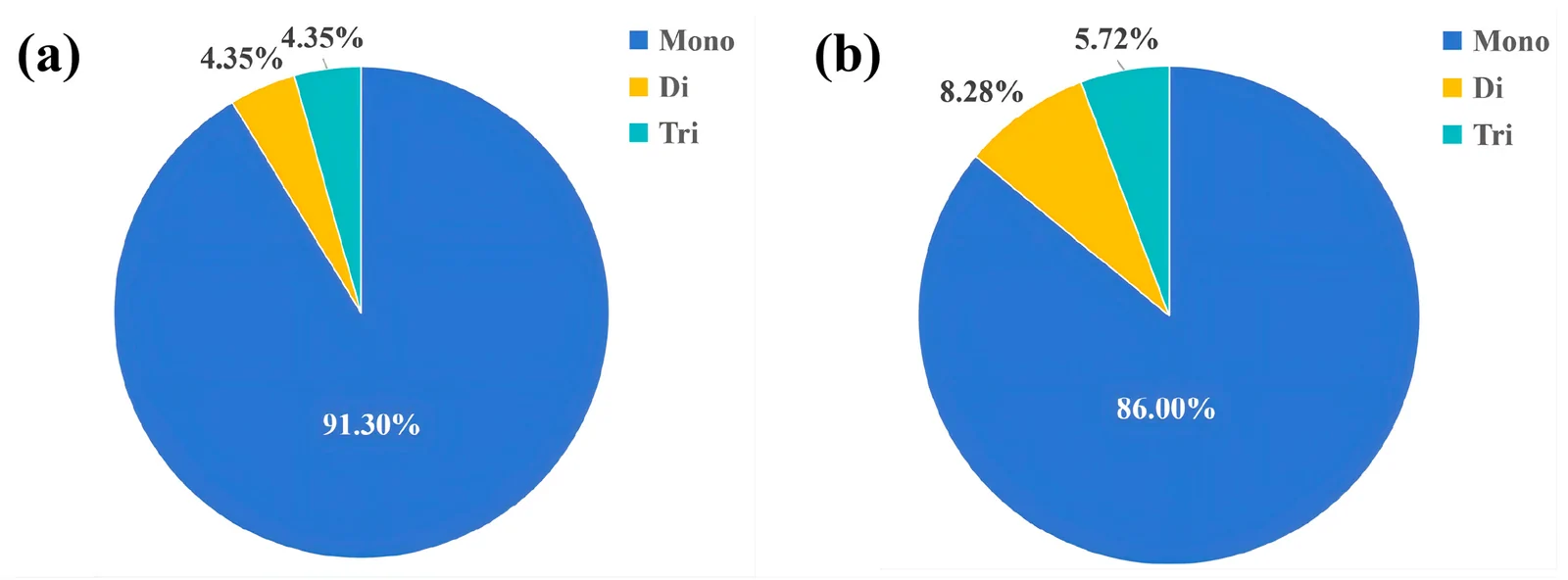
Distribution of simple sequence repeats (SSRs) in the *C. tangutica* chloroplast genome. (**a**) Counts of different SSR motif types. (**b**) Length distribution of SSR motif types (bp).

The chloroplast genome contained 23 SSRs, with 15 located in intergenic regions and 8 within coding sequences. The coding-region SSRs were restricted to five genes: *ycf1*, *psbC*, *rpoA*, *rpoB*, and *rpoC2*. Of the 23 SSRs, 21 were A-mononucleotide repeats, one was a dinucleotide (AT), and one was a trinucleotide (AAC). In terms of SSR type distribution, mononucleotide repeats accounted for 91.30% of the total SSR count, while di- and trinucleotide repeats each accounted for 4.35% (Figure 2a). In terms of total length, mononucleotide repeats contributed 270 bp (86.00%), followed by dinucleotide (26 bp, 8.28%) and trinucleotide (18 bp, 5.72%) repeats (Figure 2b). These results indicate a strong A/T bias in the SSR composition of the *C. tangutica* chloroplast genome (Table S3).

### 3.3. Comparative Genomic Analysis

When compared with eight other plastomes (six Ranunculaceae and two Paeonia), the *C. tangutica* genome showed only minor deviations in size and gene content. All nine genomes were roughly 160 kb with 38% GC. Protein-coding genes numbered 83–92, while rRNAs remained constant at eight and tRNAs at 36–39. The *C. tangutica* plastome was 135 bp larger than the MK253446.1 accession, with the LSC region accounting for most of this size difference (Table S4).

**Figure 3:**
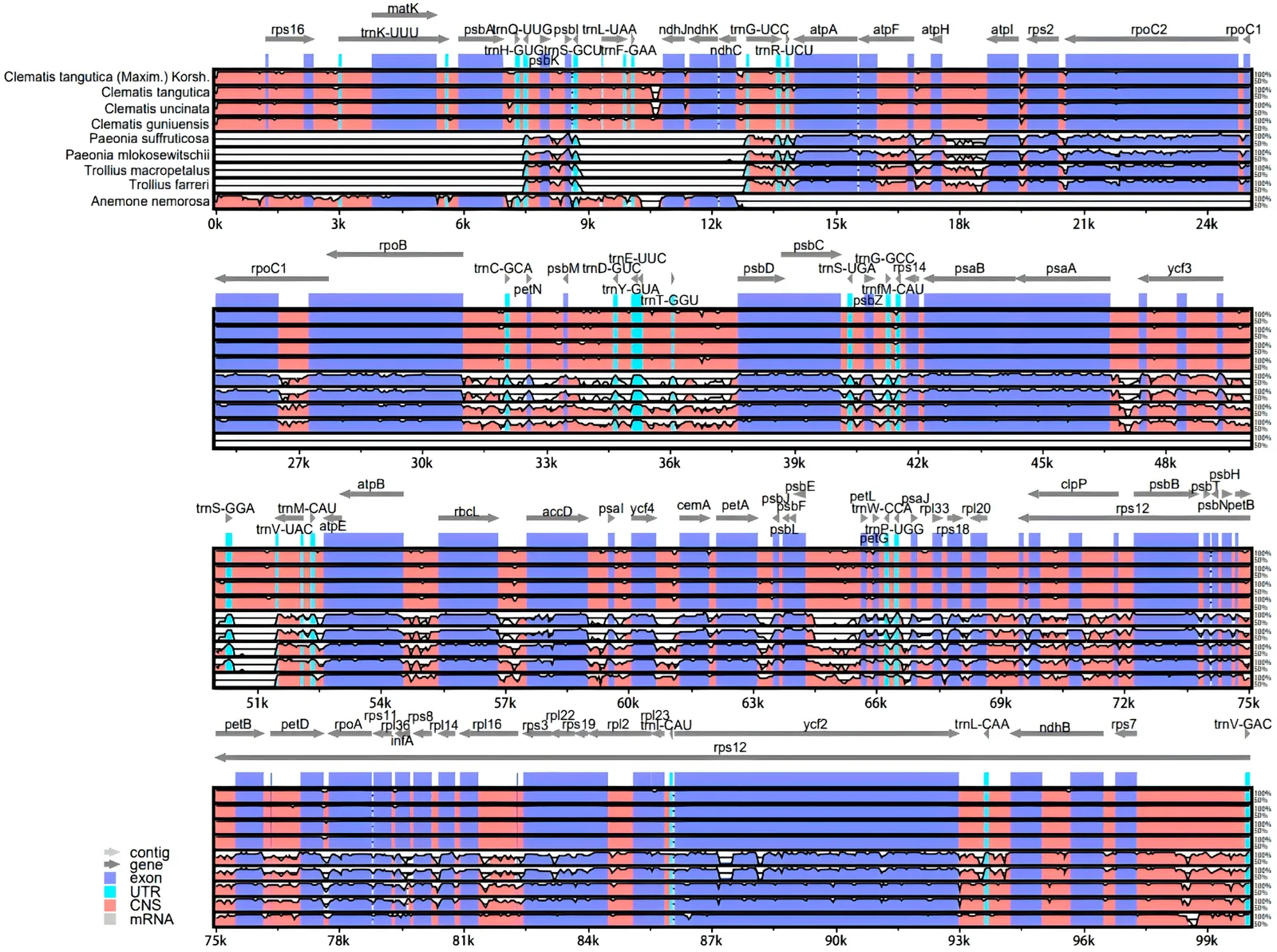
mVISTA alignment of nine Ranunculaceae chloroplast genomes. The genome of *C. tangutica* (Maxim.) Korsh. served as the reference. The horizontal axis shows the genomic position; the y-axis shows sequence identity (0–100%, with highly conserved regions above 50%). A legend at the bottom left provides the color-coding scheme for exons, UTRs, CNS, and mRNAs. Arrows above the alignment indicate the transcriptional direction of genes.

Sequence variations among the nine plastomes were detected via mVISTA. The analysis revealed that *C. tangutica* was less divergent from *C. uncinata* and *C. guniuensis*, but lacked several structural regions found in *C. florida* (Figure 3).

**Figure 4:**
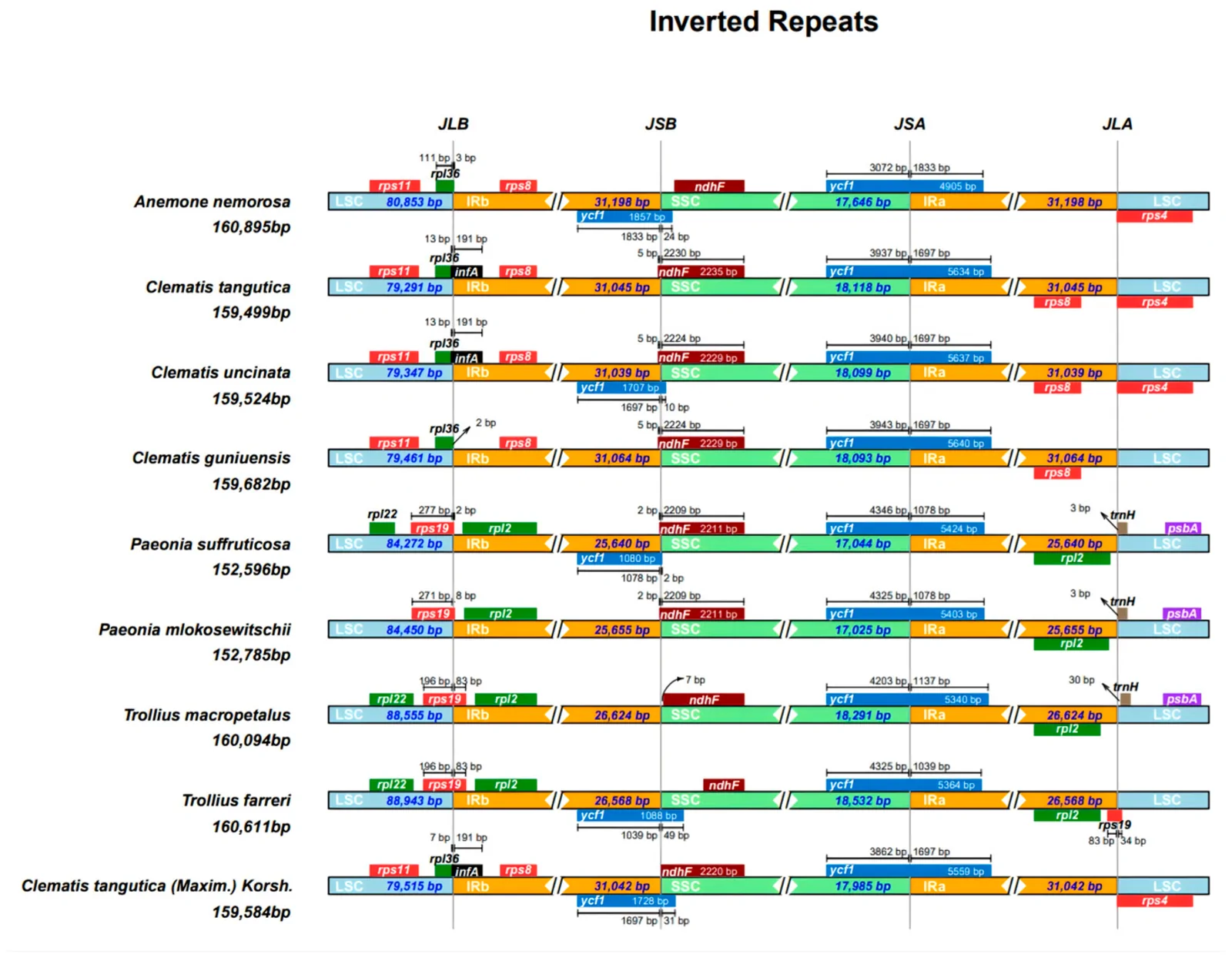
LSC, SSC, and IR junction comparison across nine Ranunculaceae plastomes. The four junctions (JLB, JSB, JSA, JLA) are indicated. Gene names and distances (bp) from the junctions are shown. Boxes represent genes, with their lengths drawn to scale. SSC, LSC, IR regions are labeled. IRb/SSC junction (JSB) is relatively conserved across all species. The *ycf1* gene at this boundary shows a striking variation, shifting from over 1600 bp in *Anemone* and *Clematis* species to only 2–49 bp in *Paeonia*, *Trollius*, and other *Clematis* lineages. In addition, significant IR expansions are observed at the JLA (SSC/IRa) boundary in *Paeonia* and *Trollius* species, resulting in the incorporation of the *trnH* and *psaA* genes into the IR regions.

Chloroplast genome alignment using mVISTA showed that genic regions exhibited higher conservation than intergenic regions., and that *C. tangutica* exhibited relatively low sequence divergence from *C. uncinata* and *C. guniuensis* (Figure 4). Analysis of IR boundaries revealed that the IRb/SSC junction was the most conserved across all nine species, while the position of the *ycf1* locus at the IRb/SSC junction varied from 2 to 49 bp among species.

**Figure 5:**
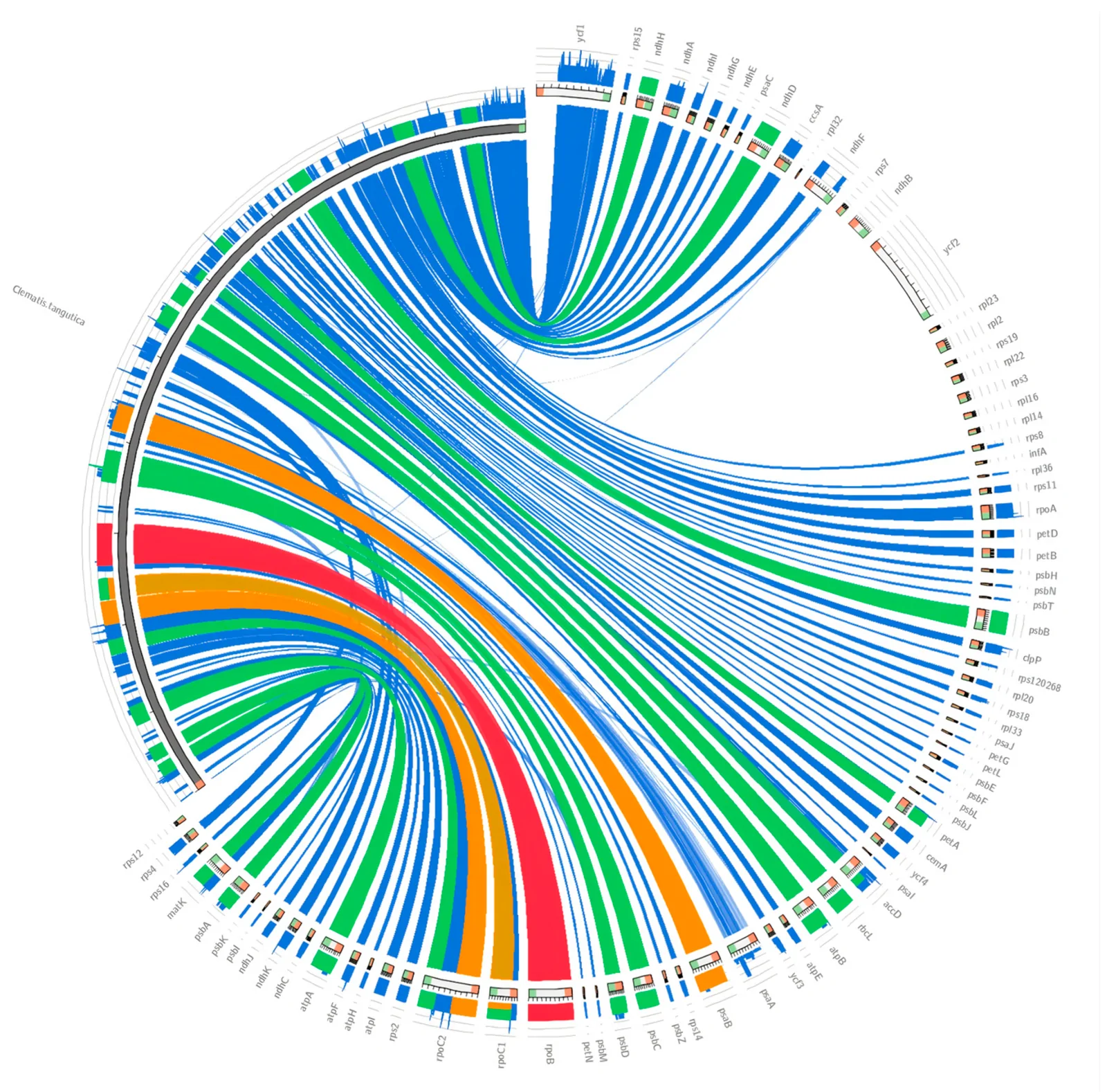
Comparison of chloroplast genome sequence similarity between *C. tangutica* and eight other Ranunculaceae species. The outer ring represents the genomic positions of the compared species, labeled with gene names. The colored lines in the inner ring represent the alignment of homologous blocks, with red, orange, green, and blue indicating sequence blocks of long, medium, relatively short, and short lengths, respectively. The links connect regions of high sequence similarity between the genomes. This figure visualizes the degree of sequence divergence between *C. tangutica* and the other eight species, with thicker and warmer-colored links generally indicating longer conserved genomic blocks, while the dense blue lines highlight the highly conserved inverted repeat (IR) regions.

**Figure 6:**
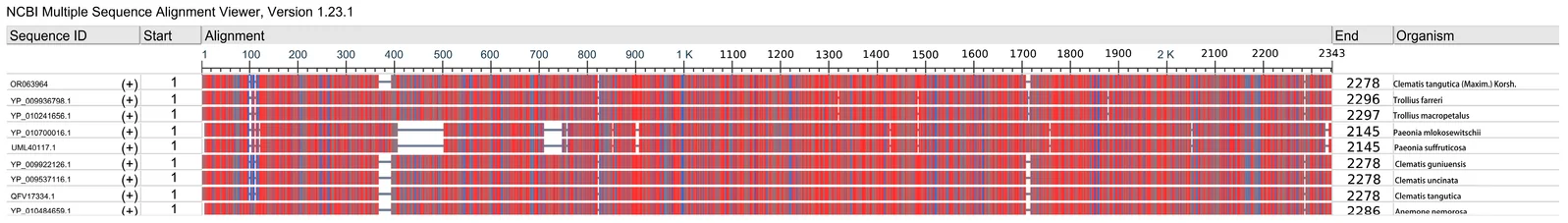
Comparison of the *ycf2* protein sequences between *C. tangutica* and eight other Ranunculaceae species. The aligned amino acid sequences are shown with different colors representing distinct amino acid properties. Gaps (white spaces) indicate sequence deletions. Four Clematis species share a 26-amino acid deletion in the region spanning positions 360–383, while *Paeonia mlokosewitschii* and *Paeonia suffruticosa* share a larger deletion spanning positions 400–500. The alignment reveals that the *ycf2* protein of *C. tangutica* shows the highest similarity to those of *C. guniuensis* and *C. uncinata* among the eight other species.

Thirteen gene loci (*rps7*, *ndhB*, *ycf2*, *rpl23*, *rpl2*, *rps19*, *rpl22*, *rps3*, *rpl16*, *rpl14*, *rps8*, *infA*, and *rps12*) exhibited sequence divergence among the nine species. Among these, *ycf2* showed the most pronounced variation (Figure 5). Alignment of the *ycf2* protein sequences revealed that four Clematis species shared a 26-amino acid deletion in the region spanning positions 360–383, while *P. mlokosewitschii* and *P. suffruticosa* shared a 94-amino acid deletion in the 400–500 region (Figure 6). The *ycf2* gene of *C. tangutica* showed the highest similarity to those of *C. guniuensis*, *C. uncinata*, and *C. tangutica* (MK253446.1).

### 3.4. Phylogenetic Tree Construction

**Figure 7:**
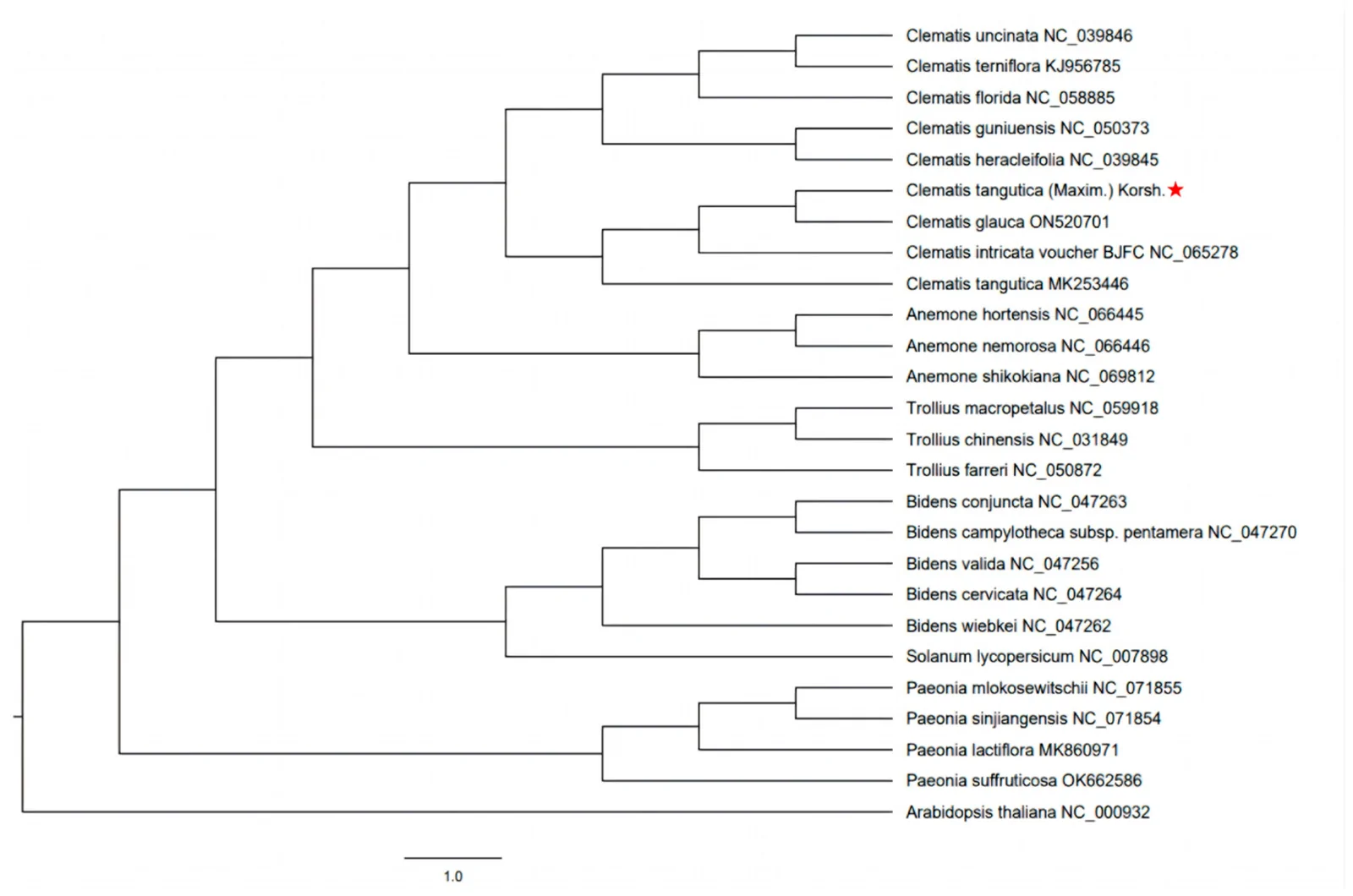
Phylogenetic tree inferred from 26 complete chloroplast genomes using maximum likelihood. The branch lengths are proportional to the number of substitutions per site (scale bar at the bottom). *C. tangutica* (indicated with a red star, ★) is most closely related to *C. glauca*. All included Ranunculaceae species form a distinct clade, with *Arabidopsis thaliana* located at the base.

To place *C. tangutica* phylogenetically, we built an ML tree from 26 complete chloroplast genome sequences, including 17 Ranunculaceae species (representing *Clematis*, *Trollius*, and *Anemone*) and nine outgroup taxa (*Paeonia spp.*, *Arabidopsis thaliana*, *Solanum lycopersicum*, and five *Bidens species*) (Figure 7). The ML tree revealed that all Ranunculaceae species formed a well-supported monophyletic clade, which was further divided into four major subclades. Within the *Clematis clade*, *C. tangutica* was most closely related to *C. glauca*, with strong bootstrap support (100%). The remaining Clematis species included *C. uncinata*, *C. terniflora*, *C. florida*, *C. guniuensis*, *C. heracleifolia*, *C. intricata*, and *C. tangutica* (MK253446.1). The outgroup taxa (*Bidens*, *Solanum*, *Paeonia*, and *Arabidopsis*) were positioned outside the Ranunculaceae clade, consistent with their phylogenetic placement.

### 3.5. Positively Selected Sites in ycf2

Positive selection in the *ycf2* gene was tested in nine Ranunculaceae species using EasyCodeML v1.2 with codon-based models. LRTs supported M2a and M8 over M1a and M7/M8a (*p* < 0.01; Table S5), indicating ω > 1 at some codons. Using NEB and BEB approaches, we identified 12 sites with posterior probabilities > 95% that were consistently supported by both methods: 103L, 352L, 356S, 362S, 363D, 366P, 368C, 369L, 374R, 376F, 377T, and 382Q. These 12 sites are all located in the middle region of the *ycf2* protein (approximately positions 350–382).

## 4. Discussion

### 4.1. General Features of the C. tangutica Chloroplast Genome

The length of *C. tangutica* complete chloroplast genome is 159,584 bp, with a typical four-part structure and a gene content (134 genes) that falls within the range observed in other Ranunculaceae species. Its overall GC content (37.9%) is similar to those of related taxa, and the higher GC content in the IR region (42.0%) versus the LSC (36.3%) and SSC (31.4%) regions reflects the presence of four GC-rich rRNA genes, a pattern commonly observed in angiosperm chloroplast genomes [37]. The 17 intron-containing genes, including the trans-spliced *rps12*, are largely consistent with those found in other Clematis species [38], indicating that the chloroplast genome of *C. tangutica* is generally conserved in structural organization.

In chloroplast genomes, substitution rates vary across genomic regions; they display higher sequence conservation than non-coding regions and SCs, which is attributable to functional constraints and copy-dependent repair pathways [39,40]. This differential mutation dynamic is consistent with our observation that coding regions and IR boundaries are more conserved than non-coding regions and SC regions (Figure 4). These genomic features confirm the reliability of our assembly and provide a foundation for subsequent analyses.

### 4.2. Repeat Sequences and SSR Markers

A total of 23 SSRs were identified in the *C. tangutica* chloroplast genome, most of which are mononucleotide repeats (21 out of 23) composed of adenine, indicating a strong A/T bias consistent with observations in other angiosperm chloroplast genomes [13]. Notably, 15 of the 23 SSRs are located in non-coding regions, whereas only eight are found in coding regions (distributed in *ycf1*, *psbC*, *rpoA*, *rpoB*, and *rpoC2*). This distribution pattern supports the notion that non-coding regions accumulate more repeats due to lower selective constraints [41]. The SSR markers will serve as useful tools for future population genetics, biogeography, and conservation studies in *C. tangutica* and related species.

### 4.3. ycf2 as a Hotspot of Sequence Variation

Comparative analysis across nine Ranunculaceae chloroplast genomes revealed that *ycf2* exhibits the highest sequence divergence among the 13 variable loci identified (Figure 6), consistent with reports in *Orchidaceae* [20] and *Asteraceae* [21]. Notably, we observed a 26-amino acid deletion in the *ycf2* protein (positions 360–383) specifically shared among four *Clematis* species (Figure 7), suggesting that this deletion may serve as a lineage-specific marker for the genus *Clematis*. In contrast, *P. mlokosewitschii* and *P. suffruticosa* exhibited a larger deletion spanning positions 400–500, implying lineage-specific evolutionary dynamics of *ycf2* across different families.

The high variability of *ycf2* aligns with its emerging role as a valuable phylogenetic marker. Previous studies have demonstrated that *ycf2* alone can produce phylogenetic trees largely congruent with those based on whole chloroplast genome data, owing to its long sequence length and relatively slow substitution rate [42]. Thus, *ycf2* may offer a cost-effective alternative for inferring phylogenetic relationships in species-rich groups when whole-plastome sequencing is not feasible.

### 4.4. Adaptive Evolution of ycf2

We identified 12 positively selected sites clustered in the central region of the *ycf2* protein (positions 350–382), which overlaps with the 26-amino acid deletion observed in *Clematis* species. Although these sites show signatures of positive selection, their functional consequences remain unknown without protein structure data or experimental validation.

Recent structural studies have identified *ycf2* as the core ATPase motor of the chloroplast protein import machinery [19]. The Qinghai–Tibet Plateau is characterized by high UV radiation, low temperatures, and low oxygen, conditions that stress the photosynthetic apparatus. The positive selection signals in *ycf2* may therefore reflect adaptive adjustments in protein import efficiency, consistent with reports of positive selection in *ycf2* in other lineages [20,21], suggesting a conserved role in environmental adaptation across angiosperms.

Positive selection in chloroplast genes has been increasingly reported in high-altitude plants. In Rhodiola [43] and Meconopsis [44], chloroplast genes involved in photosynthesis and protein import were found to be positively selected, linking plastid evolution to adaptation to low CO_2_ and cold conditions. The positive selection signals in ycf2 are consistent with this broader pattern, suggesting that adjustments in protein import efficiency may contribute to high-altitude adaptation.

Site-directed mutagenesis has been successfully applied to validate positive selection sites in chloroplast genes, like the *ndhF* gene in orchids [16] and the *rbcL* gene in Helianthus [21]. Following these approaches, the functional significance of the 12 sites identified in *ycf2* could be tested by individually mutating each site and assessing its effects on ATPase activity and protein–protein interactions of the Ycf2-FtsHi complex.

Nevertheless, several limitations should be acknowledged. Expanding taxonomic sampling beyond the nine Ranunculaceae species analyzed here would increase statistical power, and direct correlations with environmental variables (e.g., altitude, UV intensity) would strengthen the adaptive hypothesis. Future studies integrating environmental data and protein structural modeling are needed to establish a direct link between the observed positive selection signals and high-altitude adaptation. Despite these limitations, this study provides the first evidence of adaptive evolution of *ycf2* in Ranunculaceae and highlights its potential role in high-altitude adaptation.

## 5. Conclusions

In our study, we assembled the first complete chloroplast genome of *C. tangutica*, a Tibetan medicinal plant from the Qinghai–Tibet Plateau. The genome is 159,584 bp in length, contains 134 genes, and harbors 23 SSRs with a strong A/T bias, providing useful markers for future population studies. Comparative analyses with eight other Ranunculaceae species revealed that the chloroplast genomes are generally conserved, whereas the *ycf2* gene exhibits the highest sequence divergence. Notably, we detected 12 positive selection sites in *ycf2*, all clustered in the middle region of the protein, suggesting that this gene may have been subject to adaptive evolution. This study provides the first genomic resource for *C. tangutica* and offers new insights into the adaptive evolution of *ycf2* in Ranunculaceae.

## Figures and Tables

**Table 1:** Summary of chloroplast genome assembly statistics for *C. tangutica.*

Parameter	Value	Parameter	Value
Genome feature		Gene content	
Total genome size (bp)	159,584	Total genes	134
LSC (bp)	79,515	Protein-coding genes	88
SSC (bp)	17,985	tRNA genes	38
IR (bp, each)	31,042	rRNA genes	8
Overall GC content (%)	37.9	IR boundary junctions (bp)	
GC in LSC (%)	36.3	JLB (LSC/IRb)	79,516
GC in SSC (%)	31.4	JSB (IRb/SSC)	110,557
GC in IR (%)	42.0	JSA (SSC/IRa)	128,543
		JLA (IRa/LSC)	159,584

## Data Availability

The assembled data is available at NCBI: OR063964.

## Supplementary Materials

The following supporting information can be downloaded at https://www.mdpi.com/article/10.3390/genes17080930/s1: Table S1: List of genes in the chloroplast genome of *C. tangutica (Maxim.) Korsh.*; Table S2: Characteristics of genes including introns and exons in the chloroplast genome of *C. tangutica (Maxim.) Korsh.*; Table S3:The abundance of each standard motif category; Table S4: Comparison of chloroplast genome features among nine Ranunculaceae species; Table S5: Likelihood ratio statistics (2ΔlnL) for pairwise model comparisons.

## References

[1] Moon A.E.R., Han J.E., Lee B.Y., Park J.M., Jang C.G.E. (2013). An Unrecorded Species of *Genus clematis* (Ranunculaceae) from Korea. J. Asia-Pac. Biodivers..

[2] Wang R.B., Ni W.Y., Xu W.J., Gui Z.W., Zhou S.B.A.O. (2019). *Clematis guniuensis* (Ranunculaceae), a new species from Eastern China. PhytoKeys.

[3] Zeng Y.X., Zhao C.X., Liang Y.Z., Yang H., Fang H.Z., Yi L.Z., Zeng Z.D. (2007). Comparative analysis of volatile components from Clematis species growing in China. Anal. Chim. Acta.

[4] Guo X., Wang G., Li J., Li J., Sun X. (2023). Analysis of Floral Color Differences between Different Ecological Conditions of *Clematis tangutica* (Maxim.) Korsh. Molecules.

[5] Zhang H., Guo X., Wei X., Wang L., Zhong Q., Sun X. (2025). Potential Distribution Prediction and Metabolite Analysis of *Clematis tangutica* (Maxim.) Korsh. On the Qinghai Plateau. Ecol. Evol..

[6] Zhang X., Ren Q., Guo X., Chen Y., Sun S., Wang M., Ren X., Liu C., Zhang M. (2026). *Clematis tangutica* extract alleviates hyperlipidemia: An integration of network pharmacology, molecular docking, molecular dynamics and experimental verification. J. Ethnopharmacol..

[7] Yang Z. (2007). PAML 4: Phylogenetic Analysis by Maximum Likelihood. Mol. Biol. Evol..

[8] Asaf S., Khan A.L., Khan M.A., Waqas M., Kang S.M., Yun B.W., Lee I.J. (2017). Chloroplast genomes of *Arabidopsis halleri* ssp. gemmifera and *Arabidopsis lyrata* ssp. petraea: Structures and comparative analysis. Sci. Rep..

[9] Kadam S.K., Youn J.S., Kim J.H. (2026). Refined chloroplast annotations, repeat profiles, and phylogenomic evidence reveal maternal lineage shifts and independent evolution in the Triticum-Aegilops complex. BMC Plant Biol..

[10] Santos L.A. (2025). Beyond conservation: The landscape of chloroplast genome rearrangements in angiosperms. New Phytol..

[11] Daniell H., Lin C.S., Yu M., Chang W.J. (2016). Chloroplast genomes: Diversity, evolution, and applications in genetic engineering. Genome Biol..

[12] Cauz-Santos L.A., Costa Z.P., Sader M.A., van den Berg C., Vieira M.L.C. (2025). Chloroplast genomic insights into adaptive evolution and rapid radiation in the genus *Passiflora* (Passifloraceae). BMC Plant Biol..

[13] Chen Y., Liu N., Wang H., Luo H., Xie Z., Yu H., Chang Y., Zheng B., Zheng X., Sheng J. (2025). Comparative chloroplast genomics of rutaceae: Structural divergence, adaptive evolution, and phylogenomic implications. Front. Plant Sci..

[14] Li C.C., Bao Y., Hou T., Li J.C., Ma Z.Y., Wang N., Wu X.M., Xie K.D., Zhou Y.F., Guo W.W. (2024). Insights into chloroplast genome evolution in Rutaceae through population genomics. Hortic. Adv..

[15] Gao L.Z., Liu Y.L., Zhang D., Li W., Gao J., Liu Y., Li K., Shi C., Zhao Y., Zhao Y.J. (2019). Evolution of Oryza chloroplast genomes promoted adaptation to diverse ecological habitats. Commun. Biol..

[16] Liu Y., Zhang Q., Wu Z., Shi Z., Wang S. (2026). Chloroplast Genome Evolution in Pleurothallidinae (Orchidaceae): Lineage-Specific Selection, Codon Usage Patterns, and Phylogenetic Implications. Genes.

[17] Drescher A., Ruf S., Calsa T., Carrer H., Bock R. (2000). The two largest chloroplast genome-encoded open reading frames of higher plants are essential genes. Plant. J..

[18] Liang K., Zhan X., Li Y., Yang Y., Xie Y., Jin Z., Xu X., Zhang W., Lu Y., Zhang S. (2024). Conservation and specialization of the Ycf2-FtsHi chloroplast protein import motor in green algae. Cell.

[19] Liang K., Jin Z., Zhan X., Li Y., Xu Q., Xie Y., Yang Y., Wang S., Wu J., Yan Z. (2024). Structural insights into the chloroplast protein import in land plants. Cell.

[20] Li Z.H., Ma X., Wang D.Y., Li Y.X., Wang C.W., Jin X.H. (2019). Evolution of plastid genomes of *Holcoglossum* (Orchidaceae) with recent radiation. BMC Evol. Biol..

[21] Zhong Q., Yang S., Sun X., Wang L., Li Y. (2019). The complete chloroplast genome of the Jerusalem artichoke (*Helianthus tuberosus* L.) and an adaptive evolutionary analysis of the ycf2 gene. PeerJ.

[22] Wu Z., Liao R., Yang T., Dong X., Lan D., Qin R., Liu H. (2020). Analysis of six chloroplast genomes provides insight into the evolution of *Chrysosplenium* (Saxifragaceae). BMC Genom..

[23] Shi C., Hu N., Huang H., Gao J., Zhao Y.J., Gao L.Z. (2012). An Improved Chloroplast DNA Extraction Procedure for Whole Plastid Genome Sequencing. PLoS ONE.

[24] Luo R., Liu B., Xie Y., Li Z., Huang W., Yuan J., He G., Chen Y., Pan Q., Liu Y. (2012). SOAPdenovo2: An empirically improved memory-efficient short-read de novo assembler. Gigascience.

[25] Bankevich A., Nurk S., Antipov D., Gurevich A.A., Dvorkin M., Kulikov A.S., Lesin V.M., Nikolenko S.I., Pham S., Prjibelski A.D. (2012). SPAdes: A New Genome Assembly Algorithm and Its Applications to Single-Cell Sequencing. J. Comput. Biol..

[26] Swain M.T., Tsai I.J., Assefa S.A., Newbold C., Berriman M., Otto T.D. (2012). A post-assembly genome-improvement toolkit (PAGIT) to obtain annotated genomes from contigs. Nat. Protoc..

[27] Lowe T.M., Eddy S.R. (1997). tRNAscan-SE: A program for improved detection of transfer RNA genes in genomic sequence. Nucleic. Acids. Res..

[28] Greiner S., Lehwark P., Bock R. (2019). OrganellarGenomeDRAW (OGDRAW) version 1.3.1: Expanded toolkit for the graphical visualization of organellar genomes. Nucleic. Acids. Res..

[29] Dubchak I., Ryaboy D.V. (2006). VISTA Family of Computational Tools for Comparative Analysis of DNA Sequences and Whole Genomes. Methods in Molecular Biology.

[30] Amiryousefi A., Hyvönen J., Poczai P. (2018). IRscope: An online program to visualize the junction sites of chloroplast genomes. Bioinformatics.

[31] Bi G., Mao Y., Xing Q., Cao M. (2018). HomBlocks: A multiple-alignment construction pipeline for organelle phylogenomics based on locally collinear block searching. Genomics.

[32] Katoh K., Standley D.M. (2013). MAFFT Multiple Sequence Alignment Software Version 7: Improvements in Performance and Usability. Mol. Biol. Evol..

[33] Minh B.Q., Schmidt H.A., Chernomor O., Schrempf D., Woodhams M.D., von Haeseler A., Lanfear R. (2020). IQ-TREE 2: New Models and Efficient Methods for Phylogenetic Inference in the Genomic Era. Mol. Biol. Evol..

[34] Huelsenbeck J.P., Ronquist F. (2001). MRBAYES: Bayesian Inference of Phylogenetic Trees. Bioinformatics.

[35] Gao F., Chen C., Arab D.A., Du Z., He Y., Ho S.Y.W. (2019). EasyCodeML: A visual tool for analysis of selection using CodeML. Ecol. Evol..

[36] Xu B., Yang Z. (2013). PAMLX: A Graphical User Interface for PAML. Mol. Biol. Evol..

[37] Yang Y., Dang Y., Li Q., Lu J., Li X., Wang Y. (2014). Complete chloroplast genome sequence of poisonous and medicinal plant Datura stramonium: Organizations and implications for genetic engineering. PLoS ONE.

[38] Liu S., Du W., Guo L., Lu H., Song W., Wu W. (2026). Complete chloroplast genome features and phylogenetic analysis of Clematis cadmia. Sci. Rep..

[39] Wicke S., Schneeweiss G.M., de Pamphilis C.W., Müller K.F., Quandt D. (2011). The Evolution of the Plastid Chromosome in Land Plants: Gene Content, Gene Order, Gene Function. Plant Mol. Biol..

[40] Wang J., Kan S., Liao X., Zhou J., Tembrock L.R., Daniell H., Jin S., Wu Z. (2024). Plant Organellar Genomes: Much Done, Much More to Do. Trends Plant Sci..

[41] Gandhi S.G., Awasthi P., Bedi Y.S. (2010). Analysis of SSR dynamics in chloroplast genomes of Brassicaceae family. Bioinformation.

[42] Yermagambetova M., Almerekova S., Ibraimov A., Osmonali B., Turuspekov Y., Abugalieva S. (2026). Characterization and comparative analysis of the complete chloroplast genomes of twelve Allium species from Kazakhstan. Sci. Rep..

[43] Yu X., Wei P., Chen Z., Li X., Zhang W., Yang Y., Liu C., Zhao S., Li X., Liu X. (2023). Comparative Analysis of the Organelle Genomes of Three Rhodiola Species Provide Insights into Their Structural Dynamics and Sequence Divergences. BMC Plant Biol..

[44] Zhao S., Gao X., Yu X., Yuan T., Zhang G., Liu C., Li X., Wei P., Li X., Liu X. (2024). Comparative Analysis of Chloroplast Genome of *Meconopsis* (Papaveraceae) Provides Insights into Their Genomic Evolution and Adaptation to High Elevation. Int. J. Mol. Sci..

