# Supplementary material for: The Complete Chloroplast Genome of Clematis tangutica (Maxim.) Korsh. and an Adaptive Evolutionary Analysis of the ycf2 Gene
Source: Genes (Basel). 2026 Aug 10;17(8):930. doi: 10.3390/genes17080930 (PMC13512289; doi:10.3390/genes17080930)
Supplement: Supplementary file 1 [file genes-17-00930-s001.zip › Table S1.pdf]

Table S1 List of genes in the chloroplast genome of *C. tangutica (Maxim.) Korsh.*

Category	Genes	Count
Photosynthesis-related genes		
Photosystem I	psaA, psaB, psaC, psaI, psaJ	5
Photosystem II	psbA, psbB, psbC, psbD, psbE, psbF, psbH, psbI, psbJ, psbK, psbL, psbM, psbN, psbT, psbZ	15
Cytochrome b/f complex	petA, petB, petD, petG, petL, petN	6
ATP synthase	atpA, atpB, atpE, atpF, atpH, atpI	6
NADH dehydrogenase	ndhA, ndhB(×2), ndhC, ndhD, ndhE, ndhF, ndhG, ndhH, ndhI, ndhJ, ndhK	11
Rubisco large subunit	rbcL	1
Protein synthesis-related genes		
RNA polymerase	rpoA, rpoB, rpoC1, rpoC2	4
Ribosomal proteins (SSU)	rps2, rps3(×2), rps4(×2), rps7(×2), rps8(×2), rps11, rps12(×3), rps14, rps15, rps16, rps18, rps19(×2)	12
Ribosomal proteins (LSU)	rpl2(×2), rpl14(×2), rpl16(×2), rpl20, rpl22(×2), rpl23(×2), rpl32, rpl33, rpl36	9
Transfer RNAs	trnA-UGC (×2), trnC-GCA, trnD-GUC, trnE-UUC, trnF-GAA, trnfM-CAU, trnG-GCC, trnG-UCC, trnH-GUG, trnI-CAU (×2), trnI-GAU (×2), trnK-UUU, trnL-CAA (×2), trnL-UAA, trnL-UAG, trnM-CAU, trnN-GUU (×2), trnP-UGG, trnQ-UUG, trnR-ACG (×2), trnR-UCU, trnS-GCU, trnS-GGA, trnS-UGA, trnT-GGU, trnV-GAC (×2), trnV-UAC, trnW-CCA, trnY-GUA	29
Ribosomal RNAs	rrn4.5 (×2), rrn5 (×2), rrn16 (×2), rrn23 (×2)	4
Other genes		
ATP-dependent protease subunit	clpP	1
Maturase	matK	1
Translation initiation factor	infA	1
Acetyl-CoA carboxylase	accD	1
Cytochrome c biogenesis	ccsA	1
Inner membrane protein	cemA	1
Hypothetical chloroplast reading frames		

ycf genes	ycf1 (×2), ycf2 (×2), ycf3, ycf4	4
Total	112	

Note: Counts represent the number of distinct gene types. Genes marked with (×2) or (×3) in the Genes column are present in two or three copies in the genome due to IR region duplications. The total number of gene copies (including duplicated copies) is 134, comprising 88 protein-coding genes, 38 tRNA genes, and 8 rRNA genes. Some genes (*rps12*, *clpP*, *ycf3*) contain two introns, while *rps12* is trans-spliced.
