# Supplementary material for: The Complete Chloroplast Genome of Clematis tangutica (Maxim.) Korsh. and an Adaptive Evolutionary Analysis of the ycf2 Gene
Source: Genes (Basel). 2026 Aug 10;17(8):930. doi: 10.3390/genes17080930 (PMC13512289; doi:10.3390/genes17080930)
Supplement: Supplementary file 1 [file genes-17-00930-s001.zip › Table S2.pdf]

Table S2 Characteristics of genes including introns and exons in the chloroplast genome of *C. tangutica (Maxim.) Korsh.*

Gene	Region	Exon I (bp)	Intron I (bp)	Exon II (bp)	Intron II (bp)	Exon III (bp)
rps16	LSC	40	882	221		
trnK-UUU	LSC	37	2545	35		
trnL-UAA	LSC	35	491	50		
trnG-UCC	LSC	23	717	48		
atpF	LSC	410	740	145		
rpoC1	LSC	1613	768	430		
trnV-UAC	LSC	35	583	38		
petB	LSC	6	483	642		
petD	LSC	8	731	496		
rpl16	IR	399	977	9		
rpl2	IR	434	651	391		
ndhB	IR	756	708	777		
trnI-GAU	IR	37	947	35		
trnA-UGC	IR	38	813	35		
ndhA	SSC	539	1001	553		
clpP	LSC	246	699	289	795	71
ycf3	LSC	152	743	230	726	124

Note: A total of 17 intron-containing genes were identified, including 11 protein-coding genes and 6 tRNA genes. Among them, *trnK-UUU* contains the longest intron (2,545 bp), while *petB* has the shortest (483 bp). The *clpP* and *ycf3* genes each contain two introns, whereas the other 15 intron-containing genes have a single intron. The *rps12* gene is trans-spliced and therefore not listed in this table.
