# Supplementary material for: The Complete Chloroplast Genome of Clematis tangutica (Maxim.) Korsh. and an Adaptive Evolutionary Analysis of the ycf2 Gene
Source: Genes (Basel). 2026 Aug 10;17(8):930. doi: 10.3390/genes17080930 (PMC13512289; doi:10.3390/genes17080930)
Supplement: Supplementary file 1 [file genes-17-00930-s001.zip › Table S3.pdf]

Table S3. The abundance of each standard motif category

Motif	Type	Counts	Length (bp)	Percent (%)	Average length (bp)	Relative Abundance (loci/Mb)	Relative Density (bp/Mb)
A	1	21	270	91.3	12.86	131.59	1691.9
AT	2	1	26	4.35	26.0	6.27	162.92
AAC	3	1	18	4.35	18.0	6.27	112.79
