# Supplementary material for: The Complete Chloroplast Genome of Clematis tangutica (Maxim.) Korsh. and an Adaptive Evolutionary Analysis of the ycf2 Gene
Source: Genes (Basel). 2026 Aug 10;17(8):930. doi: 10.3390/genes17080930 (PMC13512289; doi:10.3390/genes17080930)
Supplement: Supplementary file 1 [file genes-17-00930-s001.zip › Table S4.pdf]

Table S4.Comparison of chloroplast genome features among nine Ranunculaceae species.

Species	Size (bp)				G+C (%)	Total number of genes			GeneBank accessions
	Total	LSC	IR	SSC		Protein-coding genes	rRNAs	tRNAs	
C. tangutica (Maxim.) Korsh.	159584	79515	62084	17985	37.90	92	8	36	
C.tangutica	159449	79291	62090	18118	37.95	91	8	36	MK253446.1
C.uncinata	159524	79347	62078	18099	38.02	90	8	36	NC_039846.1
C. guniuensis	159682	79461	62128	18093	37.95	86	8	36	NC_050373.1
P. suffruticosa	152596	84272	51280	17044	38.37	85	8	37	OK662586.1
P. mlokosewitschii	152785	84450	51310	17025	38.43	83	8	37	NC_071855.1
T. macropetalus	160094	88555	53248	18291	38.03	87	8	39	NC_059918.1
T. farreri	160611	88943	53136	18532	38.05	87	8	36	NC_050872.1
A. nemorosa	160895	80853	62396	17646	37.66	90	8	36	NC_066446.1
