# Supplementary material for: The Complete Chloroplast Genome of Clematis tangutica (Maxim.) Korsh. and an Adaptive Evolutionary Analysis of the ycf2 Gene
Source: Genes (Basel). 2026 Aug 10;17(8):930. doi: 10.3390/genes17080930 (PMC13512289; doi:10.3390/genes17080930)
Supplement: Supplementary file 1 [file genes-17-00930-s001.zip › Table S5.pdf]

Table S5. Likelihood ratio statistics (2ΔlnL) for pairwise model comparisons.

Comparison between models	2ΔlnL	d.f.	p-value
M0 vs. M3	136.3408	4	0.0000<0.01
M1a vs. M2a	83.3817	2	0.0000<0.01
M7 vs. M8	88.7187	2	0.0000<0.01
M8a vs. M8	83.2980	1	0.0000<0.01

Note: 2ΔlnL represents the likelihood ratio test statistic, calculated as twice the difference in log-likelihoods between the alternative model and the null model. d.f. = degrees of freedom.
